# Leaf Gas Exchange and Photosystem II Fluorescence Responses to CO_2_ Cycling

**DOI:** 10.3390/plants12081620

**Published:** 2023-04-11

**Authors:** James Bunce

**Affiliations:** USDA-ARS, Adaptive Cropping Systems Laboratory, Beltsville, MD 20705, USA; buncejames49@gmail.com

**Keywords:** elevated CO_2_, fluctuation, photosynthesis, stomatal conductance, photosystem II, cycling, fluorescence

## Abstract

Experimental systems to simulate future elevated CO_2_ conditions in the field often have large, rapid fluctuations in CO_2_. To examine possible impacts of such fluctuations on photosynthesis, the intact leaves of the field-grown plants of five species were exposed to two-minute cycles of CO_2_ between 400 and 800 μmol mol^−1^, lasting a total of 10 min, with photosynthesis, stomatal conductance and PSII fluorescence measured at the end of each half-cycle and also 10 min after the end of the cycling. Prior to the cyclic CO_2_ treatments, the steady-state responses of leaf gas exchange and fluorescence to CO_2_ were determined. In four of the five species, in which stomatal conductance decreased with increasing CO_2_, the cyclic CO_2_ treatments reduced stomatal conductance. In those species, both photosynthesis and the photochemical efficiency of PSII were reduced at limiting internal CO_2_ levels, but not at saturating CO_2_. In the fifth species, there was no change in stomatal conductance with CO_2_ and no change in either photosynthesis or PSII efficiency at any CO_2_ level with CO_2_ cycling. It is concluded that in many, but not all, species, fluctuations in CO_2_ may reduce photosynthesis at low CO_2_, partly by decreasing the photochemical efficiency of photosystem II as well as by decreasing stomatal conductance.

## 1. Introduction

With the continuing increase in CO_2_ concentrations in the atmosphere [1], there has been considerable research examining the impacts of changes in CO_2_ concentration on plant functions and growth [2,3,4,5]. As a substrate for photosynthesis, CO_2_ is still currently a growth-limiting resource for plants that have C_3_ metabolism. Experiments imposing different CO_2_ concentrations on growing plants generally use CO_2_ sensors to dynamically regulate the supply of CO_2_ to the experimental system, while any removal of CO_2_ required during daylight is usually accomplished by plant photosynthesis and/or wind. Concerns over the impacts of short-term variations in CO_2_ concentration on plant function resulted primarily from the recognition of large-magnitude CO_2_ fluctuations in free-air-carbon dioxide-enrichment facilities. Free-air-CO_2_-enrichment (FACE) facilities were developed to provide elevated CO_2_ treatments to plant ecosystems outdoors with a minimal disturbance from other environmental factors, such as wind, light, air temperature and humidity, and soil conditions [6]. However, in most FACE systems, CO_2_ release is at the perimeter of the plot, while the CO_2_ concentration sampled to control the CO_2_ release is near the center of the plot, often many meters from the release points. Because air movement is needed to distribute CO_2_ across the plot, there is a variable time lag between CO_2_ release and the detection of the achieved concentration as well as the disturbance by air turbulence. A few papers documented large fluctuations in CO_2_ concentrations over time within a given plot with FACE systems, using sampling systems that averaged CO_2_ concentrations over about 5 s periods [7,8]. Surprisingly, despite the existence of rapid-response open-path CO_2_ analyzers for about the last 25 years, rapid (seconds) CO_2_ concentration measurements in FACE plots have only recently been published [9,10]. Based on measurements in a FACE system of the Brookhaven National Laboratory design, Allen et al. [10] concluded that “due to the difficulty of controlling elevated CO_2_ concentrations in turbulent air, the range of fluctuations of CO_2_ in FACE experiments are more than 10-fold greater than plants experience in natural conditions”. After reviewing experiments comparing plant responses to elevated CO_2_ with different degrees of fluctuation, it was concluded that plant growth was suppressed by the larger CO_2_ fluctuations in FACE systems, probably by reducing photosynthesis [10].

Because of the difficulty of reproducing fluctuations observed in FACE plots in controlled experiments, most experiments to assess the impacts of fluctuating CO_2_ have used either regular cycles of CO_2_ or brief pulses of high CO_2_ [11,12,13,14,15]. Hendrey et al. [11] measured chlorophyll fluorescence responses to the short-term cyclic variation in CO_2_ concentration of several frequencies. Holtum and Winter [12] measured responses of CO_2_ uptake to the short-term cyclic variation in CO_2_ concentration but did not measure stomatal conductance, and found that variations in CO_2_ reduced photosynthesis in two tree species. Bunce [13] provided long-term cyclic CO_2_ treatments compared with constant elevated CO_2_ treatments at the same mean elevated CO_2_ in open top chambers, and found that the cyclic CO_2_ treatments reduced photosynthesis, stomatal conductance and plant growth in wheat and cotton. Short-term series of pulses of elevated CO_2_ mimicking those observed in FACE plots reduced photosynthesis and stomatal conductance in wheat and rice leaves [14]. In indoor chambers, a larger magnitude of continuously applied fluctuations of CO_2_ reduced photosynthesis, stomatal conductance and the growth of four herbaceous species compared with a smaller amplitude of CO_2_ variation [15]. Although reduced stomatal conductance often occurs in response to CO_2_ fluctuations, it is not the sole cause of reductions in photosynthesis, even if the stomatal closure is entirely “patchy” in nature [15,16].

This work examined whether a reduced photochemical efficiency of photosystem II occurred in response to CO_2_ fluctuations and might cause some of the suspected reductions in photosynthesis in field-grown plants in FACE systems, in addition to reductions in stomatal conductance.

## 2. Results

**Figure 1:**
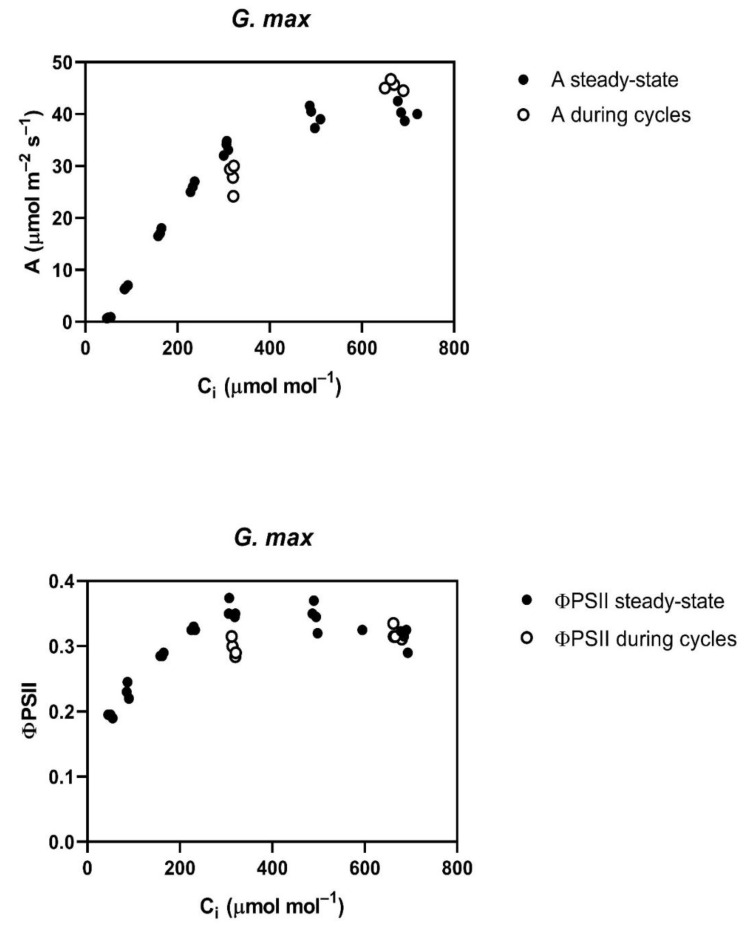

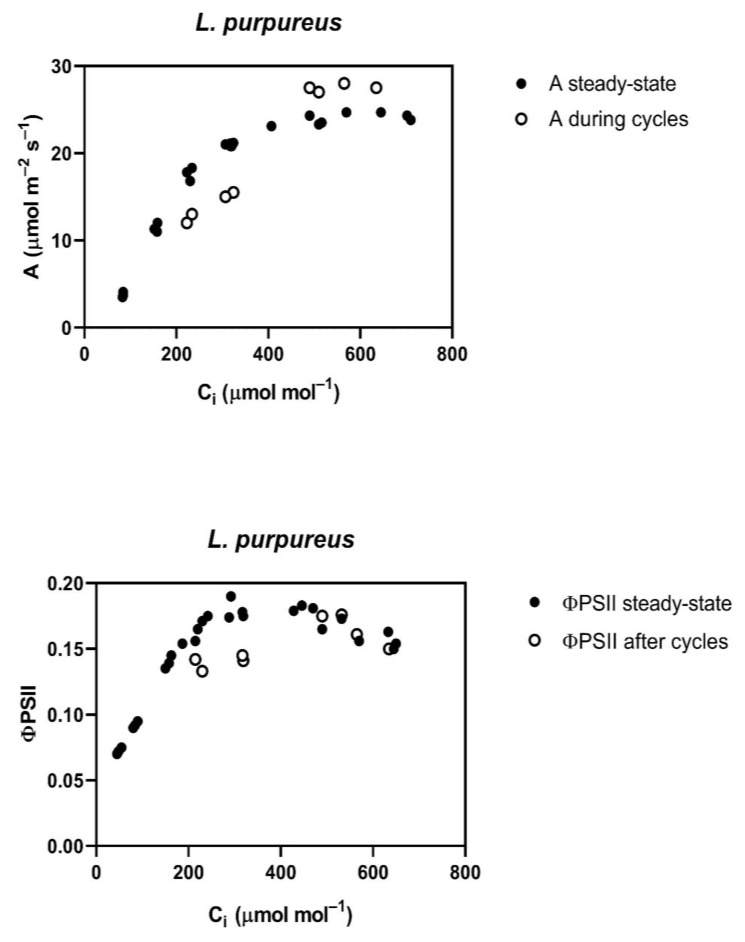

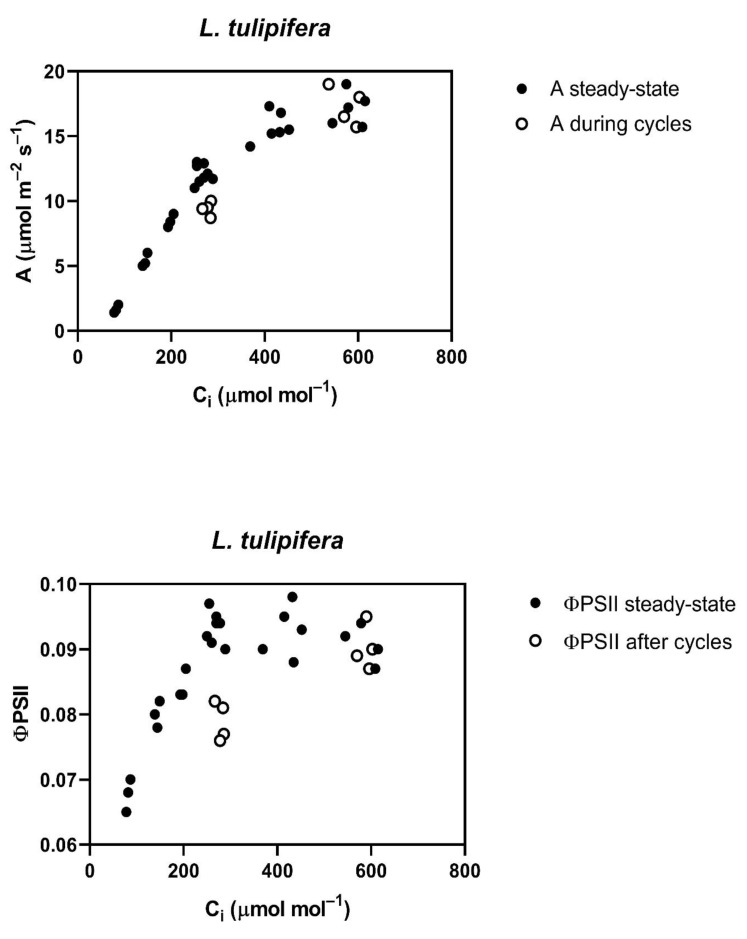

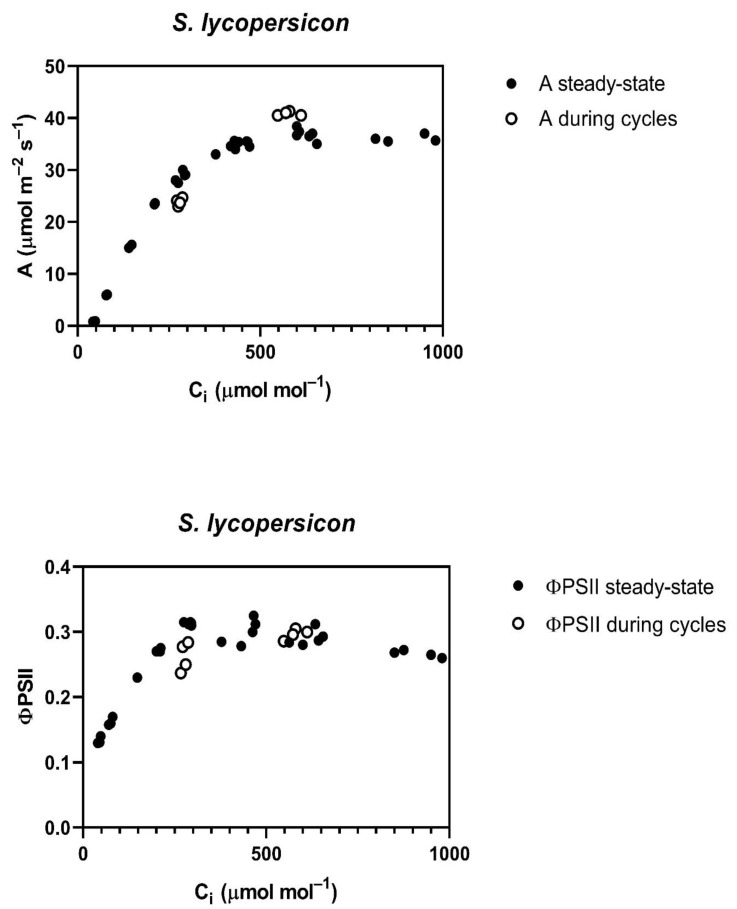
Responses of CO_2_ assimilation rate (A) and PSII efficiency (ΦPSII) as a function of sub-stomatal CO_2_ (C_i_) before (steady-state) and during cycling of ambient CO_2_ in four species. CO_2_ was cycled between 400 and 800 μmol mol^−1^, with one minute at each concentration before changing to the other concentration, for a total of 10 min. Each data point represents a measurement on a different plant taken after values had stabilized during the cycling.

Throughout the cycling of CO_2_, four of the five species studied, *G. max*, *L. purpureus, L. tulipifera* and *S. lycopersicum,* had a reduced assimilation rate (A) and PSII efficiency (ΦPSII) at rate-limiting sub-stomatal CO_2_ (C_i_) values of about 250 to 300 μmol mol^−1^ occurring at 400 μmol mol^−1^ external CO_2_ (Figure 1). At a higher C_i_, occurring at 800 μmol mol^−1^ external CO_2_, A was actually slightly increased in all of these species, except *L. tulipifera*, and the ΦPSII was the same as before the cycling of CO_2_ in all four of these species (Figure 1). The reduction in ΦPSII and A to below steady-state values was evident at the end of the first 400 μmol mol^−1^ half-cycle and continued throughout the cycling of CO_2_ in all of these four species. In *G. max*, the stomatal conductance decrease caused by cycling was nearly complete in the first half-cycle, while the other species had slower decreases in stomatal conductance, but stomatal conductance had stabilized before the end of the 10 min of cycling. All species were the same as *G. max* in terms of the speed of the ΦPSII decrease, i.e., it decreased by the end of the first half-cycle. The decrease in ΦPSII during CO_2_ cycling, observed at the lower C_i_, was accompanied by increased non-photochemical quenching. Ten minutes after the end of CO_2_ cycling, a lower stomatal conductance remained at each CO_2_ level in all four of these species (Table 1). Additionally, at ten minutes after the end of CO_2_ cycling, ΦPSII and photosynthesis measured at 400 μmol mol^−1^ both remained lower than before the CO_2_ cycling. However, the values of A and ΦPSII measured at 600 μmol mol^−1^ did not differ significantly from control values when measured at 600 μmol mol^−1^ (Table 2) in any species, despite a lower stomatal conductance in all species except *P. crispum*.

**Figure 2:**
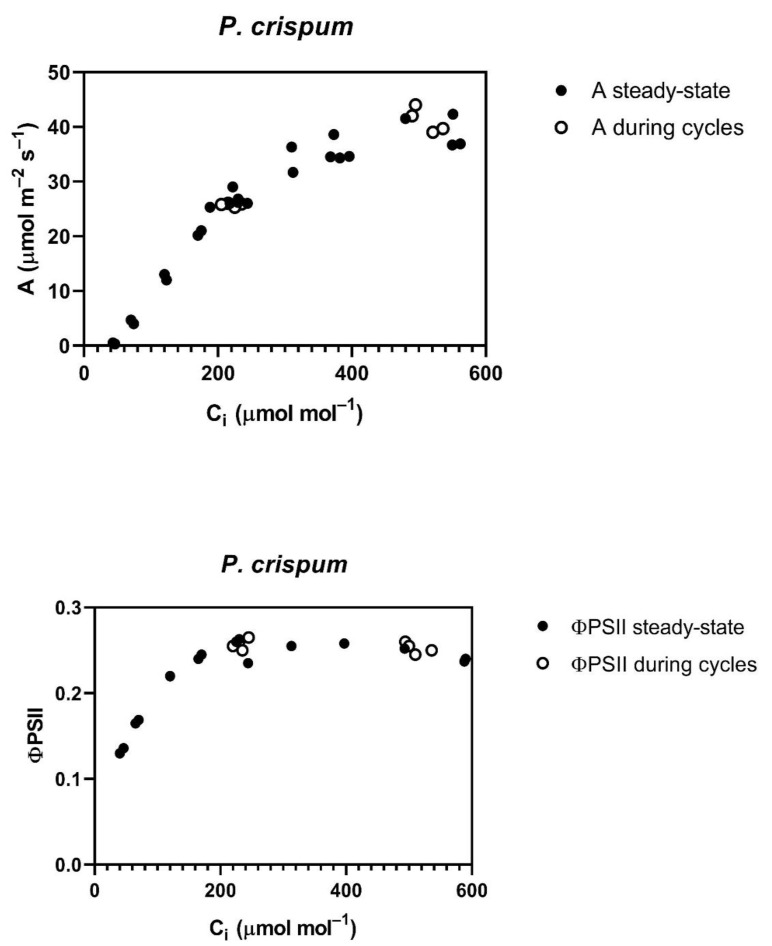
Responses of CO_2_ assimilation rate (A) and PSII efficiency (ΦPSII) as a function of sub-stomatal CO_2_ (C_i_) before (steady-state) and during cycling of ambient CO_2_ in *P. crispum*. CO_2_ was cycled between 400 and 800 μmol mol^−1^, with one minute at each concentration before changing to the other concentration, for a total of 10 min. Each data point represents a measurement on a different plant taken after values had stabilized during the cycling.

The *P. crispum*, in contrast to the other four species, had no reduction in the A vs. C_i_ curve, or in ΦPSII after the cycling of CO_2_ (Figure 2), and also showed no change in stomatal conductance with CO_2_ (Table 1).

Stomatal conductance before the cycling of CO_2_ was lower at 800 than at 400 μmol mol^−1^ CO_2_ in all species except *P. crispum* (Table 1). Stomatal conductance during CO_2_ cycling was reduced in all species, except *P. crispum* (Table 1). Ten minutes after cycling ended, the stomatal conductance remained lower than before cycling in all species, except *P. crispum*, in which the stomatal conductance was unchanged by all treatments (Table 1).

## 3. Discussion

All of these species had fairly typical A vs. C_i_ curves for C_3_ species, with no decreases in A at the highest C_i_ values, which would be clear evidence of a limitation by triose phosphate utilization (TPU) [17]. However, all species had some decrease in ΦPSII at the highest C_i_ values, which McClain et al. [18] suggest is indicative of TPU limitation. A premature leveling off of A vs. C_i_ curves is more difficult to discern than reductions in ΦPSII as an indication of TPU limitation, except possibly by the fitting of a photosynthesis model that includes a TPU limitation to the observed data.

The reductions in the photochemical efficiency of PSII (ΦPSII) at 400 μmol mol^−1^ external CO_2_ levels caused by the cycling of CO_2_ concentration, which occurred in four of the five species examined, provide a new explanation of reduced photosynthesis rates for a given sub-stomatal CO_2_ concentration, which has frequently been reported in CO_2_ fluctuation experiments [12,13,14,15]. Prior suggestions that reduced photosynthesis might be the result of “patchy” stomatal closure [13,15] admittedly could not account for the lack of reduction in photosynthesis at elevated measurement CO_2_ [15]. In the current experiments, the reduction in ΦPSII that occurred at the lower measurement CO_2_ did not occur at the higher measurement CO_2_. At the higher measurement CO_2_, photosynthesis was also not inhibited by the cycling of CO_2_ in these experiments, despite the continued lower stomatal conductance. The lack of decrease in A despite a lower stomatal conductance is to be expected at nearly saturating values of CO_2_. Similar to the results presented here, in long-term cyclic CO_2_ exposures in open top chambers, the relative reductions in photosynthesis in cotton were much larger for measurements made at the lower (near-ambient CO_2_) than at the higher external CO_2_ of the cycles [13].

McClain et al. [18] also reported reductions in ΦPSII in response to a large step increase in CO_2_, which they proposed was related to a triose-phosphate limitation of photosynthesis at high CO_2_. They provided no information on the stomatal conductance response to their treatments. However, in the fluctuating CO_2_ experiments reported here, reduced ΦPSII only occurred at limiting CO_2_ concentrations, not at elevated CO_2_. This difference in plant response might be related to the much shorter duration of exposure to high CO_2_ and lower elevated CO_2_ concentrations in the present experiment (800 μmol mol^−1^) compared with those of McClain et al. (1500 μmol mol^−1^). In these experiments, leaves were actually at 800 μmol mol^−1^ during the cycling of CO_2_ for less than five minutes.

I speculate that *P. crispum* had a qualitatively different photosynthetic response to the cyclic CO_2_ treatment than the other four species studied here, because it had no response at all of stomatal conductance to CO_2_ in the range of 400 to 800 μmol mol^−1^, in contrast to all of the other species. Similar results for more species with stomates unresponsive to changes in CO_2_ would be required to confirm this correlation. *L. tulipifera* was chosen for these experiments, based on the generally smaller response of stomatal conductance to CO_2_ in tree species [19,20]. It did have a smaller relative response than the other three herbaceous species, but not a zero response, as occurred in *P. crispum*. It remains unclear how the presence or absence of changes in stomatal conductance during fluctuations in CO_2_ could influence photochemical limitations on photosynthesis at low CO_2_. However, the decrease in photosynthesis and ΦPSII observed in this tree species at the lower measurement CO_2_ is consistent with the decreases in photosynthesis found by Holtum and Winter in two tropical tree species [12]. This suggests that FACE experiments may also not give the most accurate indication of tree responses to climate change.

Allen et al. [10] reviewed yield data in FACE and open top chambers (OTC) for several major C_3_ crop species, and they concluded that the yield stimulation caused by the same elevated CO_2_ treatments was, in FACE, on average, only about 0.66× of that occurring in OTC. A smaller yield stimulation by elevated CO_2_ in FACE than in OTC was documented for wheat and soybeans in the only side-by-side simultaneous FACE and OTC comparisons of crop yield [21] that exist to date. Allen et al. [10] tentatively attributed this smaller yield stimulation to a reduced stimulation of photosynthesis by elevated CO_2_ in FACE than in OTC. The smaller stimulation of photosynthesis was thought to be caused by the much larger fluctuations in CO_2_ in elevated CO_2_ treatments in FACE than in OTC. Allen et al. [10] carefully documented larger CO_2_ fluctuations in FACE with all of the available rapid CO_2_ measurement data, and I am not aware of any more recent published data on CO_2_ fluctuations in FACE. However, at the time that paper was written [10], reasons why rapid fluctuations in CO_2_ would cause reduced photosynthesis were unclear, despite some documented cases of high-CO_2_ pulses or the cycling of CO_2_ reducing photosynthesis [12,13,14]. Deceases in photosynthesis caused by the pulses of elevated CO_2_ or by the cycling of CO_2_ have now been documented in many of the most important C_3_ crop species, wheat [14], rice [14], soybean ([15] and this paper), and cotton [14], in two minor crop species, tomato and lablab [this paper], and also in three tree species ([12] and this paper). Up until the current work, the only clue about the reasons why fluctuations in CO_2_ would inhibit photosynthesis were observations of a reduced stomatal conductance to water vapor [13,14,15].

The results presented here provide a new mechanism by which fluctuations in CO_2_ around leaves can inhibit photosynthesis, a decrease in the photochemical efficiency of photosystem II. Of course, these results beg the question of why ΦPSII was decreased by the cycling of CO_2_. Furthermore, the extent to which this decrease in ΦPSII at low CO_2_ occurs in experiments exposing plants to a long-term elevation of CO_2_, for example in FACE experiments, has not been determined. It is interesting to consider that reduced photosynthesis in FACE systems may primarily occur during those periods in which CO_2_ fluctuations bring CO_2_ levels down to near-ambient CO_2_ levels, based on the results presented here. Most measurements of photosynthesis in FACE systems have been conducted at the targeted elevated CO_2_ concentration, not at lower CO_2_ concentrations. The only experiment to date that directly compared photosynthesis in plants grown simultaneously at elevated CO_2_ in open top chambers and in FACE systems only measured leaf gas exchange at the elevated CO_2_ [21], in the plants grown at elevated CO_2_, and thus would have missed photosynthetic responses resembling those presented here.

## 4. Materials and Methods

Leaf gas exchange and chlorophyll fluorescence measurements were conducted on four species of herbaceous plants and one tree species grown outdoors at ambient CO_2_. The species studied were *Glycine max* L. Merr. cv. Clark, *Lablab purpureus* L. Sweet, *Petroselinum crispum* Mill. Fuss var. *neopolitanum*, *Solanum lycopersicum* L. cv. Better Boy, and *Lireodendron tulipifera* L. The four herbaceous species were grown in Annapolis, Maryland in an unshaded plot with a sandy loam soil. Plants were grown from seed and planted in late April 2020. The plot was fertilized with a complete fertilizer containing 12% N, 4% P, and 8% K at 200 g of fertilizer per m^2^, and it did not experience soil water stress. The *L. tulipifera* trees sampled were saplings, about 6 years old, growing at a south-facing forest edge in Annapolis, on a sandy loam soil. Leaf gas exchange and chlorophyll fluorescence measurements were conducted from mid-June through to the end of June 2020. The mean temperature in Annapolis in May 2020 was 16.0 °C, slightly below the long-term mean of 17.7 °C, and in June 2020 it was 23.3 °C, which equals the long-term mean temperature.

All leaf gas exchange and chlorophyll fluorescence measurements were conducted at 27 °C leaf temperature, 1500 μmol m^−2^ s^−1^ PPFD, with a leaf-to-air water vapor pressure difference of 1 to 1.5 kPa, using a Ciras-3 portable photosynthesis system with a PLC3 leaf chamber/fluorometer, with an air flow rate of 400 cm^3^ min^−1^. The “stored differential balance” function of the instrument was used to correct measurements for changes in calibration with background CO_2_. The values of sub-stomatal CO_2_ (C_i_) were calculated from photosynthesis, stomatal and boundary layer conductances, and external CO_2_ by the system software. During the mornings of sunny days, a fully expanded upper-canopy leaf was selected for measurement. Steady-state responses of stomatal conductance, photosynthesis, and PS II chlorophyll fluorescence at CO_2_ concentrations of 400, 600, and 800 μmol mol^−1^ were determined on a leaf, allowing sufficient time for the stomatal conductance to adjust to each CO_2_ level, as observed on the graphical display of incoming data. Steady-state values were used to ensure that C_i_ values were accurate. The efficiency of PSII was assessed using multipulse fluorescence measurements at each CO_2_ level. The CO_2_ concentration was then returned to 400 μmol mol^−1^, and cycles of CO_2_ from 400 to 800 μmol mol^−1^ with a total cycle length of 2 min were then applied for 10 min, that is, one minute at 400 μmol mol^−1^, one minute at 800 μmol mol^−1^, one minute at 400 μmol mol^−1^, etc., for a total of 10 min. Photosynthesis, stomatal conductance, and PSII efficiency were recorded at the end of each half-cycle. At the end of the cyclic CO_2_ treatment, CO_2_ was returned to 400 μmol mol^−1^, and beginning ten minutes after the end of the CO_2_ cycling, photosynthesis, stomatal conductance, and PSII efficiency were measured at 400, 600, and 800 μmol mol^−1^ CO_2_. These measurements were made on at least four different plants of each species. On a few different leaves of each species, the responses of stomatal conductance, photosynthesis, and PS II chlorophyll fluorescence to CO_2_ concentrations from 100 to 1200 μmol mol^−1^ were determined. There were nine steps of CO_2_ (400, 300, 200, 100, 400, 600, 800, 1000, 1200 μmol mol^−1^). Leaves were kept at each step of CO_2_ for three to four minutes, waiting for the leaf gas exchange to stabilize, before measuring the photochemical efficiency of PSII using a multipulse measurement at each step in CO_2_. The leaf-to-air water vapor pressure difference changed by less than 10% of its initial value of 1 to 1.5 kPa during the cycling of CO_2_, which would have a minimal impact on the stomatal conductance.

## Figures and Tables

**Table 1:** Mean values of stomatal conductance measured at 400 and 800 μmol mol^−1^ CO_2_ before, during, and 10 min after cycling of CO_2_ between 400 and 800 μmol mol^−1^, with a full cycle length of 2 min, for a total of 10 min, in five species. Within rows, numbers followed by different letters are different at *p* = 0.05, using repeated measures ANOVA.

Species	Stomatal Conductance (mmol mol^−1^)					
		Before Cycling		During Cycling	After Cycling	
	CO_2_ (μmol mol^−1^):	400	800	Both CO2s	400	800
*G. max*		1643 a	1465 b	1168 c	956 d	808 e
*L. purpureus*		652 a	437 b	269 c	280 c	240 d
*L. tulipifera*		205 a	183 b	159 c	152 c	144 c
*S. lycopersicum*		797 a	638 b	493 c	537 c	531 c
*P. crispum*		313 a	310 a	315 a	322 a	316 a

**Table 2:** Means values of A and ΦPSII at 600 μmol mol^−1^ CO_2_ before and 10 min after the end of cycling of CO_2_ between 400 and 800 μmol mol^−1^ CO_2_ for 10 min. Within rows, numbers followed by different letters are different at *p* = 0.05, using repeated measures ANOVA.

Species	A (μmol m^−2^ s^−1^)		ΦPSII	
	Before	After	Before	After
*G. max*	37.1 a	36.5 a	0.333 a	0.313 a
*L. purpureus*	22.9 a	21.7 a	0.175 a	0.165 a
*L. tulipifera*	16.3 a	15.2 a	0.095 a	0.094 a
*S. lycopersicum*	34.5 a	33.7 a	0.310 a	0.308 a
*P. crispum*	35.1 a	35.3 a	0.255 a	0.257 a

## Data Availability

Data are available from the author upon request.
